# Insights into the genetic diversity of indigenous goats and their conservation priorities

**DOI:** 10.5713/ajas.18.0737

**Published:** 2019-02-09

**Authors:** Gang Liu, Qianjun Zhao, Jian Lu, Feizhou Sun, Xu Han, Junjin Zhao, Haiyong Feng, Kejun Wang, Chousheng Liu

**Affiliations:** 1 National Center for Preservation and Utilization of Animal Genetic Resources, National Animal Husbandry Service, Beijing 100193,China; 2 Institute of Animal Sciences, Chinese Academy of Agricultural Sciences, Beijing 100193, China; 3 College of Animal Science and Veterinary Medicine, Henan Agricultural University, Zhengzhou 450002, China

**Keywords:** Chinese Indigenous Goats, Genetic Diversity, Conservation Priorities

## Abstract

**Objective:**

An experiment was conducted to evaluate genetic diversity of 26 Chinese indigenous goats by 30 microsatellite markers, and then to define conservation priorities to set up the protection programs according to the weight given to within- and between-breed genetic diversity.

**Methods:**

Twenty-six representative populations of Chinese indigenous goats, 1,351 total, were sampled from different geographic regions of China. Within-breed genetic diversity and marker polymorphism were estimated calculating the mean number of alleles, observed heterozygosities, expected heterozygosities, fixation index, effective number of alleles and allelic richness. Conservation priorities were analyzed by statistical methods.

**Results:**

A relatively high level of genetic diversity was found in twenty-four population; the exceptions were in the Daiyun and Fuqing goat populations. Within-breed kinship coefficient matrices identified seven highly inbred breeds which should be of concern. Of these, six breeds receive a negative contribution to heterozygosity when the method was based on proportional contribution to heterozygosity. Based on Weitzman or Piyasatian and Kinghorn methods, the breeds distant from others i.e. Inner Mongolia Cashmere goat, Chengdu Brown goat and Leizhou goat obtain a high ranking. Evidence from Caballero and Toro and Fabuel et al method prioritized Jining Gray goat, Liaoning Cashmere goat, and Inner Mongolia Cashmere goat, which agree with results from Kinship-based methods.

**Conclusion:**

Conservation priorities were determined according to multiple methods. Our results suggest Inner Mongolia Cashmere goat (most methods), Jining Gray goat and Liaoning Cashmere goat (high contribution to heterozygosity and total diversity) should be prioritized based on most methods. Furthermore, Daiyun goat and Shannan White goat also should be prioritized based on consideration of effective population size. However, if one breed can continually survive under changing conditions, the straightforward approach would be to increase its utilization and attraction for production via mining breed germplasm characteristics.

## INTRODUCTION

Goats, one of the most ancient livestock species, were domesticated ~10,000 years ago in Southwestern Asia [1]. It is widely accepted that Chinese indigenous goat breeds originated from the plateau of Southwest China and nearby regions in central Asia. About 39% of documented mammalian livestock breeds are goats, but 7% of known goat breeds are extinct and 20% are threatened. Long-term natural and artificial selection, imposed by environmental changes and animal husbandry, have resulted in 58 indigenous and 8 cultivated goat breeds. A considerable number of desirable traits occur in Chinese indigenous and domesticated goats, such as extensive adaptability to stressful environments, outstanding disease and cold resistance, strong coarse fodder resistance, and high prolificacy. These traits are most likely multigenic and therefore represent a diverse natural gene pool. Interesting examples include the Jining gray goat (JNQ, associated with genes which determine the color of lambskin) and the Zhongwei goat (ZWS), the only fur-bearing goat in the world (associated with genes responsible for the unique fur phenotype). This genetic resource is invaluable for future goat breeding and utilization.

Recently, Chinese indigenous goat breeds have been threatened by the introduction of exotic goat breeds, which have typically been selected for optimal production of meat, wool, or other products. Because short-term profit maximization incentivizes the replacement or crossbreeding of Chinese indigenous goat breeds with more productive breeds, the indigenous gene pool is at risk. Both population size reduction and genetic variation dilution are major threats. There has been an increasing tendency to neglect the unique qualities and variety offered by indigenous breeds in favor of exotic breeds and their crosses. However, it is now recognized that it is important to establish conservation priorities and strategies to conserve genetic diversity within- and between-breeds, primarily to avoid further losses of genetic resources. Genetic diversity is one the most important factors that determines whether a breed survives and flourishes, or ultimately faces depression and extinction. The genetic diversity of domestic animals is crucial to meet current production needs in various environments, prospective and changing breeding objectives, and sustained genetic improvement. In response, various conservation strategies have been implemented. However, in order to make the best use of limited conservation funds, it is necessary to prioritize specific indigenous breeds for conservation [2].

Several conservation approaches have focused on breeds with maximum conservation value (i.e., breeds with high levels of genetic variation) [3,4]. The Weitzman approach, which has been widely employed to establish conservation priorities, uses between-breed genetic diversity to prioritize breeds that are highly distant from others based on genetic distance [4,5]. However, the Weitzman method has been criticized because it does not take within-breed diversity into consideration [5,6]. Other factors, including rare alleles that occur at anomalously high frequency due to inbreeding, strict genetic isolation, or founder effects, can compromise the effectiveness of the Weitzman method [7]. Another approach relies primarily on within-breed genetic diversity, which is usually calculated in terms of average expected heterozygosity (H_E_). The strategy prioritizes a breed if its removal from a population results in a maximum loss of global average H_E_. However, the drawback to this method is that it is insensitive to between-breed genetic diversity. Alternative methods that consider between- and within-breed diversity, based on co-ancestry or kinship, have been developed to prioritize breeds for conservation [8,9]. Although this approach ideally includes molecular and pedigree data, pedigree information is not available for many breeds, and thus in practice the method mainly depends on molecular data. To combine between- and within-breed diversity, various weights can be assigned to balance the two components [5,10–12] F_ST_ (Wright’s fixation index in total population) can be used to adjust the between-breed diversity derived from the Weitzman method, and 1-F_ST_ to adjust the within-breed diversity derived from the H_E_ change in a metapopulation [5]. For example, Piyasatian and Kinghorn assigned between-breed components five times more weight than within-breed components [11]. Ginja et al [13] and Cañón et al [14] conducted conservation priority analyses for cattle using different methods. No one method has yet emerged as the best, but together they provide a comprehensive view of genetic diversity that can inform a conservation program.

Conservation priorities have been studied using the methods described above for domestic animals, including cattle [14], pig [10], sheep [15], chicken [16], horse [17], and goat. However, priorities for Chinese indigenous goat breeds have not been examined, except for one study in which the Weitzman approach was used to rank 12 mutton goat breeds [18]. In the work presented here, we conducted a comprehensive analysis of conservation priorities for 26 Chinese indigenous goat breeds, based on 30 microsatellite markers.

## MATERIALS AND METHODS

### Animal care

Protocols for blood and tissue sampling were approved by the Biological Studies Animal Care and Use Committee of the National Animal Husbandry Services, Beijing, People’s Republic of China. All experimental procedures followed guidelines established under the Law of Animal Husbandry in the People’s Republic of China (No. IASCAAS-AE-03,12 Dec 2016). The experimental procedure was approved by the Institutional Animal Care and Committee at National Animal Husbandry Service.

### Sampling

In total, 1,351 samples from 26 representative Chinese indigenous goat breeds (Supplementary Figure S1) were used in this study. Goats from 19 provinces or autonomous regions in China were sampled, covering high altitude regions and plains. The blood samples were collected at major production centers meeting the characteristics and features of the breed, from healthy animals that were unrelated within three generations.

Thirty microsatellite markers recommended by the International Society for Animal Genetics (ISAG)/Food and Agriculture Organization of the United Nations (FAO) were used to genotype the 1,351 samples. The markers are *CSRD247*, *ETH10*, *MAF209*, *OARAE54*, *SRCRSP15*, *SRCRSP3*, *SRCRSP5*, *TGLA53*, *DRBP1*, *ILSTS087*, *INRABERN172*, *MAF065*, *MCM 527*, *OARFCB20*, *SPS113*, *SRCRSP8*, *ILSTS029*, *INRA023*, *INRA063*, *INRABERN185*, *P19(DYA)*, *SRCRSP23*, *SRCRSP9*, *TCRVB6*, *BM6444*, *ILSTS011*, *ILSTS005*, *SRCRSP7*, *OARFCB48*, and *MAF70*. DNA was isolated from cryopreserved blood samples using the DNeasy Blood and Tissue Kit (Qiagen, Valencia, CA, USA). Polymerase chain reaction (PCR) amplifications were conducted using fluorescence-labelled primers. PCR products were analyzed by capillary gel electrophoresis using an ABI-PRISM 3130xl Genetic Analyzer (Applied Biosystems, Foster City, CA, USA) and LIZ 500 internal size standards (Applied Biosystems, USA). Data were collected and analyzed using GENEMAPPER 3.5 (Applied Biosystems, USA).

### Genetic diversity analysis

Within-breed genetic diversity and marker polymorphism were estimated using GENALEX [19] and the Excel Microsatellites Toolkit to calculate the mean number of alleles (MNA), observed heterozygosities (H_O_) and H_E_. Allelic richness (R_t_) over all loci for each breed was computed with FSTAT v 2.9.3.2 [20]. In this study, estimation of N_E(0.05)_ were performed with linkage disequilibrium method and random mating model (minor allele frequency 0.05) using LDNe program [21].

The methods of conservation analyses used in this study have been demonstrated previously [13,14]. To better understand the statistical results, three approaches were employed: i) a method designed to minimize the overall kinship coefficient (Core Set method); ii) a method that considered between-breed diversity alone (Weitzman approach); iii) a method aimed at combining the within- and between-breed components of overall genetic diversity. These are described in detail below.

### Core Set methods

The Core Set method [22] is based on measures of co-ancestry or kinship, and aims to eliminate the genetic overlap between populations in a core set. The coefficient of kinship was used as a measure of genetic similarity [22]. Negative contribution estimates were avoided through an iterative process that assigns the lowest value as zero, and recalculates contributions after a removal of the population. A coefficient matrix of kinships was estimated using three methods to correct for alleles identical: i) marker-estimated kinships (MEK), in which variation is based on weighted equal drift similarity (WEDS) [23]; ii) MEK estimation based on WEDS, using a bootstrap procedure (bootstrap 250); iii) a variation of the MEK method based on log linear regression obtained with a weighted log-linear model (WLM) [24]. Analyses of conservation priorities based on similarity matrices were performed using the FORTRNA application, which was developed and kindly shared by Eding and Meuwissen [22,24].

Pairwise kinship distances were computed from the MEK matrix following Eding et al [22]. Neighbor-net phylogenies for all breeds were constructed with kinship genetic distance using SPLITS TREE soft (version 4.12.6) [25]. Average molecular co-ancestries (fm), based on allele frequency, were analyzed using MOLKIN3 [26]. Genetic relationships were used to classify the goat breeds and generate contour plots of kinship coefficients (MEK based on WEDS and *f*_m_) using the colorRamps package in R.

Within-breed contributions to diversity were computed using the average within-breed H_E_. The partial contribution made by individual breeds was computed as the proportional variation in average internal heterozygosity of the complete data set after removal of each breed (PC_He_).

### Weitzman approach

The Weitzman method was also used to calculate the partial contribution made by each breed to total genetic diversity (PC_Weitz_), based on Reynolds genetic distance (on diversity, Weitzman). This method estimates between-breed diversity and ignores within-breed diversity. Analyses conducted using the Weitzman method were computed using a FORTRAN program developed by Ollivier and Foulley [5]. Reynolds genetic distances were computed using MOLKIN3 software [26].

### Combined methods

When making decisions about conservation priority, both within- and between-breed genetic diversity should be taken into account [5]. Three approaches were used to calculate the two components of global diversity for a metapopulation: i) Ollivier and Foulley [5] proposed that the aggregate diversity (PC_Fst_) should weight the between-population diversity using the factor F_ST_, and weight the within-population diversity using the factor 1-F_ST_; ii) Piyasatian and Kinghorn proposed that the between-population diversity component should receive five times more weight than the within-population diversity (PC_5:1_) [11]; iii) Caballero and Toro [9] and Fabuel et al [10] assigned equal weights to the within-population co-ancestries and genetic distances. In this case, calculations were performed using MOLIKN3 [26]. Pairwise correlation analysis was performed using the PICANTE package of R with the Pearson method.

## RESULTS AND DISCUSSION

### Marker polymorphisms, within-breed diversity and breed relationships

Summary statistics describing microsatellite marker polymorphisms and genetic diversity per breed are presented in Table 1 and Supplementary Table S1, respectively. A total of 430 alleles were observed in 26 Chinese indigenous goat populations, with locus *BM6444* showing the largest observed (N_A_, 12.385±0.60) and effective (N_E_, 6.202±0.299) allele number (Supplementary Table S1). In contrast, the smallest values for N_A_ and N_E_ were 2.308±0.092 and 1.393±0.06 at locus *MAF209*. The mean H_O_ for all loci is 0.577±0.008, ranging from 0.269± 0.041 at locus *MAF209* to 0.785±0.031 at locus *OARFCB48*. The mean H_E_ is 0.638±0.006, ranging from 0.25±0.03 at locus *MAF209* to 0.827±0.01 at locus *BM6444*. The F_ST_ value across the 30 loci has a relatively high mean (0.141±0.010), indicating that there is genetic differentiation among the 26 Chinese indigenous goat populations. There are 23 loci exhibit positive F_IS_ values, and the positive mean F_IS_ is 0.082±0.029. This may indicate non-random mating, and these loci may be morphological or productive traits under selection.

The MNA was relatively high in the 26 Chinese indigenous goat populations, of which the largest was in the Xinjiang goat (XJS, 8.23±2.93), and the lowest in the Daiyun goat (DYS, 4.40±2.25) (Table 1). However, the highest and lowest values for the effective number of alleles were 4.369±0.299 in the JNQ and 2.424±0.217 in the DYS. The mean Rt was 5.61± 2.04, ranging from 7.03±2.27 in the XJS to 3.89±1.91 in the DYS. Additionally, higher estimates of expected and H_O_ were observed in the JNQ (H_E_, 0.737±0.024; H_O_, 0.646±0.012), whereas the values of H_E_ and H_O_ were 0.507±0.039 and 0.434± 0.013 in the DYS, representing the smallest heterozygosity (Table 1). All populations in which H_E_ exceeded H_O_ yield positive values for F_IS_.

The level of allelic diversity in this study was similar to that reported for 9 Chinese cashmere goats [27], but lower than in the Chinese Cashmere [28] and Swiss [29] goat populations. The heterozygosity of most of the breeds examined in this study was also lower than the values reported in these studies, particular the H_O_. Loss of genetic diversity among populations can be caused by genetic introgression [30], environmental change [31] and inbreeding. In our study, F_IS_ had a positive value in all breeds, demonstrating a deficit of heterozygosity (Table 1). F_IS_ is usually used to obtain a deeper understanding of the degree of endangerment and inbreeding, and is also considered as a criterion for conservation priority. In present study, 5 breeds had F_IS_ values less than 0.05; 16 breeds fell into the range 0.05 to 0.15, and 5 breeds in the range 0.15 to 0.25. These levels are higher than previously reported in Chinese cashmere goats [27]. Effective population size (N_E_) is also a valuable indictor for evaluating conservation priorities. Effective population size (N_E0.05_) were computed based on linkage disequilibrium with minor allele frequency 0.05 (Table 1). N_E0.05_ ranged from 32.7 DYS up to 569.7 ZWS. According to Franklin’s 50/500 rule of thumb [32], effective population size under 50 suggested that population is facing to serious genetic threaten. Small population are easy to inbreeding depression. The lowest population size of DYS means that it is necessary to implement conservation program. According to allele statistics, lower allele richness, H_O_, H_E_, N_EA_, MNA, and F_IS_ of Daiyun indicated a relatively high inbreeding. The reasons behind is human intervention of mating process which influencing by introduction of breeds with high performance and intensive breeding. So, DYS and Shannan White goat (SNB) (N_E0.05_ = 47.5) should be paid more attention and considered in conservation program.

**Figure 1:**
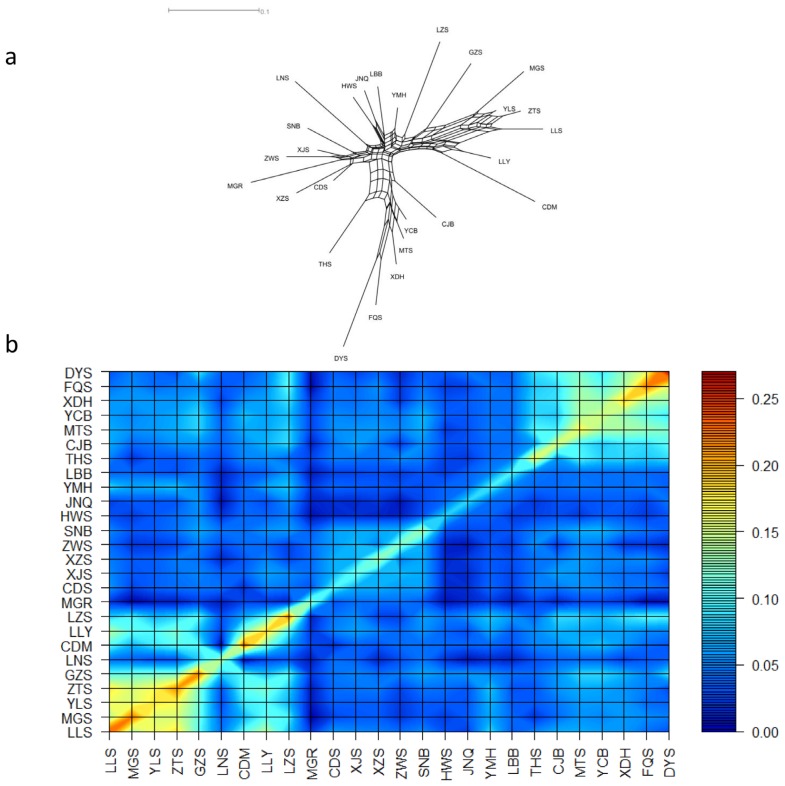
Graph representing between-breed distance and within-breed kinship. (a) Neighbor-net graph of kinship genetic distance. (b) Contour plots of marker-estimated kinships (MEK). Breed name acronyms are defined as follows: LLS, Longling yellow goat; MGS, Maguan poll goat; YLS, Yuling goat; ZTS, Zhaotong goat; GZS, Guizhou White goat; LNS, Liaoning Cashmere goat; CDM, Chengdu Brown goat; LLY, Longlin goat; LZS, Leizhou goat; MGR, Inner Mongolia Cashmere goat; CDS, Chaidamu goat; XJS, Xinjiang goat; XZS, Tibetan goat; ZWS, Zhongwei goat; SNB, Shannan White goat; HWS, Huanghuai goat; JNQ, Jining Gray goat; YMH, Yimeng Black goat; LBB, Lubei White goat; THS, Taihang goat; CJB, Yangtse River Delta White goat; MTS, Matou goat; YCB, Yichang White goat; XDH, Xiangdong Black goat; FQS, Fuqing goat; DYS, Daiyun goat.

The neighbor-net phylogeny of kinship distances shows the relationships among the 26 Chinese indigenous populations (Figure 1a). The phylogeny of kinship distances was similar to that generated using the Reynolds distance (Supplementary Figure S2a). Goats from northern China, such as the Inner Mongolia Cashmere goat (MGR), Tibetan goat, ZWS, XJS, Chaidamu goat (CDS), and SNB clustered together, and were close to the Huanghuai goat (HWS) and JNQ. Except for the Shannan white goat, these breeds belong to the North China cluster [32]. The DYS, Fuqing goat (FQS), and Xiangdong Black goat (XDH) clustered together with a long branch length, like Longlin goat (LLY) and Chengdu Brown goat (CDM) on one side, with Longling yellow goat (LLS), Zhaotong goat (ZTS), Yuling goat (YLS), and Maguan poll goat (MGS) on the other side. The Matou goat (MTS) and Yichang White goat (YCB) clustered together, partly consistent with a previous study by Wang et al [33] that placed MTS, YCB, and SNB into the Central China cluster. Although Wang et al [33] also proposed that LLS, MGS, YLS, ZTS, CDM, and LLY belonged to the Southwest China cluster, our results cluster LLY and CDM within a separate branch. The discrepancy may reflect the fact that more loci were used in the present study, supporting a more accurate analysis. The majority of goat populations cluster in agreement with their geographical origin.

Within-breed diversity could also be estimated using kinship coefficients with either the MEK derived from genotypes or the average coancestries (*f*_m_) obtained from allele frequencies. To aid in the analysis, contour plots were plotted to visualize both within- and between-breed kinships. Each population was sorted based on its genetic proximity defined in the phylogenetic neighbor-net graph (Figure 1b; Supplementary Figure S2b). The area shaded from brown to red represents highly inbred breeds: the DYS (MEK = 0.267 and *f*_m_ = 0.499), LLS (MEK = 0.242 and *f*_m_ = 0.417), CDM (MEK = 0.229 and *f*_m_ = 0.400), MGS (MEK= 0.223 and *f*_m_ = 0.408), Guizhou White goat (MEK = 0.222 and *f*_m_ = 0.424), Leizhou goat (LZS, MEK = 0.222 and *f*_m_ = 0.423), and the FQS (MEK = 0.221 and *f*_m_ = 0.436). The yellow shading represents goat breeds with intermediate kinship values: the XDH (MEK = 0.186 and *f*_m_ = 0.398), LLY (MEK = 0.185 and *f*_m_ = 0.368), YLS (MEK = 0.178 and *f*_m_ = 0.378), MTS (MEK = 0.174 and *f*_m_ = 0.381), Taihang goat (THS, MEK = 0.173 and *f*_m_ = 0.349), and the Yangtse River Delta White goat (CJB, MEK = 0.154 and *f*_m_ = 0.364). The light blue and light yellow areas also reveal goat breeds with relationships. The two groupings consist of the LLS, MGS, YLS, and ZTS (0.136<MEK<0.179, 0.348<*f*_m_<0.380), and the DYS, FQS, XDH, YCB, and MTS (0.092<MEK<0.160, 0.328< *f*_m_<0.407).

### Analyses of conservation priorities for Chinese indigenous goat

Based on different approaches, analyses of conservation priorities for Chinese indigenous goat populations is presented in Tables 2 and 3. Fourteen (out of 26) populations were estimated to make a null contribution to total genetic diversity, based on the Bootstrap, WEDS, and WLM kinship methods (Table 2). The three highly prioritized breeds were the JNQ, Liaoning Cashmere goat (LNS), and MGR (0.1361<WEDS< 0.1622, 0.1324<Bootstrap<0.1613 and 0.1071<WLM<0.2621). A similar ranking of conservation priority was obtained using all three methods. This may indicate that high within-breed genetic diversity exists in these populations.

A considerable number of populations with negative value for PC_He_ (14 breeds) were observed, based on the proportional contribution of each breed to the average H_E_ for the whole population (Table 2). For these populations, there is a gain in total diversity after they are excluded. As expected, the majority of these breeds have highly inbred status (high within-breed kinship coefficients) and relatively low heterozygosity (low H_E_) (Figure 1b; Table 1). The XDH, DYS, FQS, and MTS have the most negative PC_He_ values (−0.218<PC_He_<−0.195) (Table 2). The most important conservation priorities, ranked high to low, are the JNQ, MGR and LNS (0.258<PC_He_<0.363) based on PC_He_ value (Table 2). Breeds with intermediate PC_He_ values include the Tibet goat, HWS, Lubei White goat (LBB) and CDS (0.173<PC_He_<0.227) (Table 2).

The conservation priorities assigned using the Weitzman approach depend mainly on between-breed diversity, and therefore favor highly differentiated breeds that have large genetic distances from other breeds. The breeds making the most contribution to global diversity were the MGR, LZS, and CDM (6.57<PC_Weitz_<7). The HWS, Tibet goat, LBB and YCB (4.14<PC_Weitz_<5.6) made intermediate contributions to total diversity. The smallest contributions were made by the YLS, THS, and ZTS (1.58<PC_Weitz_<1.96) (Table 2).

Ollivier and Foulley [5] proposed that both within- and between-breed components should be taken into consideration (PC_Fst_) [5]. In this approach, the F_ST_ value of the whole populations is used to weight PC_Weitz_ (F_ST_ = 0.138 in this study) and 1-F_ST_ is used to weight PC_He_. The PC_Fst_ method prioritizes breeds by kinship (i.e., the HWS with PC_Fst_ = 0.969 and WEDS = 0.2017), and also prioritizes breeds that contribute highly to PC_Weitz_ (i.e., the CDM with PC_Fst_ = 1.034 and PC_Weitz_ = 6.71). The breed with the highest priority is the MGR (PC_Fst_ = 1.235, WEDS = 0.1622, WLM = 0.1071, PC_He_ = 0.307 and PC_Weitz_ = 7). Although the Tibet goat makes an intermediate contribution to total diversity as estimated by various methods (WEDS, WLM, PC_He_, and PC_Weitz_), it also has a relatively high PC_Fst_ value (PC_Fst_ = 0.951). However, the ranking derived from the PC_5:1_ method (as proposed by Piyasatian and Kinghorn) is similar to the priority estimated using PC_Weitz_, because it prioritizes the CDM, MGR, and LZS, due to the increased weight given to the between-breed component.

The results obtained from the combined approach proposed by Caballero and Toro [9] and Fabuel et al [10] are shown in Table 3. The breeds making the highest contributions to global coancestry were the FQS, MTS, and LZS this is the result of high within-breed coancestry (0.423<*f*_ii_<0.499) and the relatively low distance from all the other populations (0.095<D_nei_< 0.120). Although the DYS has a relatively high *f*_ii_ value (0.499), its mean genetic distance was also larger (D_nei_ = 0.147) and thus its contribution to *f* was relatively low (0.0119). The difference between *f*_ii_ and D_nei_ is responsible for calculating the contribution to global co-ancestry. The absolute contribution to total diversity prioritized the JNQ (0.0356), CD (0.0343) and Xijiang goat (0.0335). Ranking by absolute contribution yielded results similar to assigning priority based on proportional contributions to genetic diversity. Using absolute contributions, priority was assigned to the JNQ (4.808), CDS (4.632), and XJS (4.524). The ZWS has the lowest contribution (1.931), perhaps due to its relatively low sample size (n = 25). When the proportional contribution to genetic diversity was estimated without reference to sample size, only the JNQ (4.240) and CDS (4.078) maintain their higher priorities. The Tibet goat also obtained a high priority (4.051). In contrast, the DYS made the smallest contribution (PC1 = 3.052 and PC2 = 3.362). Finally, total genetic diversity was analyzed after removing one subpopulation at a time from the total population. Removal of the JNQ resulted in the largest reduction in total genetic diversity (GDT|i = 0.7378, loss/gain = −0.4%), followed by the MGR (GDT|i = 0.7382, loss/gain = −0.3%) and LNS (GDT|i = 0.7386, loss/gain = −0.3%).

Linear correlation coefficients calculated for pairwise contributions derived from different methods are shown in Table 4. No negative correlation occurs between any pairwise contributions, even between Weitzman and H_E_ contributions (0.321). The correlations between PC_5:1_ and other methods are similar to the correlation between PC_Weitz_ and others because of the excess weight given to between-breed components in PC_5:1_. Compared to PC_weighted_, higher correlations were observed between PC_unweighted_ and other methods. As there are too many null contributions in the three core set methods, it is not useful to calculate the correlation between these and the other methods, despite the higher correlation between core set methods and PC_He_.

## CONCLUSION

In conclusion, this study analyzed the extant genetic diversity of Chinese indigenous goats in terms of within- and between-breed components. Various approaches utilizing these factors were compared to evaluate their utility and shortcomings in a conservation program. Our results suggest MGR (most methods), JNQ and LNS (high contribution to heterozygosity and total diversity) should be prioritized based on above results. Furthermore, DYS and SNB also should be prioritized based on consideration of effective population size. However, if one breed could survive in changing conditions all the time, the straightforward approach is to increase its utilization and attraction for production via mining breed germplasm characteristic.

## Supplementary Data



## Figures and Tables

**Table 1:** Genetic diversity estimated using 30 microsatellite loci in each of 26 goat populations

Population1)	MNA±SD	N_EA_±SD	H_E_±SD	H_O_±SD	R_t_±SD	F_IS_	N_E(0.05)_
LLS	6.00±2.56	2.932±0.315	0.588±0.030	0.491±0.013	5.22±2.13	0.167	120.8
MGS	5.33±1.95	2.847±0.189	0.598±0.033	0.573±0.013	4.7±1.56	0.043	74.1
YLS	6.00±2.68	3.162±0.261	0.628±0.031	0.552±0.013	5.36±2.16	0.123	171.3
ZTS	5.93±2.52	2.88±0.225	0.593±0.034	0.530±0.013	5.17±1.98	0.107	97.1
GZS	5.63±2.27	2.77±0.227	0.582±0.032	0.546±0.013	4.91±1.88	0.062	113.6
LNS	6.47±2.74	3.528±0.245	0.664±0.034	0.619±0.012	5.72±2.26	0.066	153.7
CDM	5.93±2.41	2.877±0.2	0.606±0.028	0.595±0.012	5.11±1.79	0.018	80.4
LLY	5.63±2.22	3.131±0.216	0.64±0.026	0.600±0.013	5.13±1.82	0.062	74.4
LZS	5.47±2.26	2.793±0.219	0.583±0.034	0.539±0.012	4.71±1.77	0.075	75.9
MGR	6.93±2.63	3.664±0.293	0.661±0.037	0.556±0.013	6.19±2.3	0.161	143.1
CDS	7.90±3.08	4.102±0.291	0.709±0.029	0.653±0.011	6.87±2.47	0.080	124.1
XJS	8.23±2.93	3.962±0.29	0.689±0.036	0.616±0.012	7.03±2.27	0.108	101.2
XZS	7.37±3.10	3.625±0.239	0.685±0.027	0.612±0.012	6.61±2.49	0.108	96.1
ZWS	6.87±2.69	3.903±0.303	0.694±0.035	0.622±0.018	6.8±2.66	0.106	569.7
SNB	6.50±2.33	3.685±0.264	0.684±0.028	0.600±0.012	5.86±1.98	0.121	47.5
HWS	7.37±2.28	4.294±0.312	0.729±0.025	0.588±0.013	6.75±1.96	0.195	113.2
JNQ	7.30±2.51	4.369±0.299	0.737±0.024	0.646±0.012	6.56±2.12	0.124	98.3
YMH	7.83±2.77	3.859±0.299	0.704±0.023	0.557±0.013	6.73±2.24	0.210	66.1
LBB	7.67±2.51	3.822±0.234	0.707±0.025	0.628±0.013	6.72±2.07	0.113	76.7
THS	6.47±2.34	3.344±0.237	0.657±0.027	0.615±0.011	5.59±1.95	0.022	134.7
CJB	5.77±2.22	3.275±0.26	0.644±0.029	0.623±0.014	5.32±1.97	0.032	374.2
MTS	5.37±2.09	3.014±0.224	0.625±0.025	0.569±0.012	4.78±1.82	0.091	69.4
YCB	5.93±2.32	3.25±0.23	0.655±0.024	0.623±0.012	5.19±1.88	0.050	100.1
XDH	5.37±2.24	2.912±0.225	0.608±0.030	0.549±0.013	4.74±1.81	0.097	96.8
FQS	4.90±2.40	2.723±0.247	0.569±0.034	0.475±0.012	4.30±1.93	0.168	89
DYS	4.40±2.25	2.424±0.217	0.507±0.039	0.434±0.013	3.89±1.91	0.146	32.7
Average	6.33±2.47	-	0.644±0.030	0.577±0.013	5.61±2.04	0.167	-

Summary statistics of the genetic diversity in 26 goat breeds.

*n*, sample size; MNA, mean number of alleles; SD, standard deviation; N_EA_, mean number of effective alleles; H_E_, expected heterozygosity; H_O_, observed heterozygosity; R_t_, allelic richness; F_IS_, fixation index; N_E(0.05)_, effective population size based on linkage disequilibrium (minor allele frequency 0.05).

1) LLS, Longling yellow goat; MGS, Maguan poll goat; YLS, Yuling goat; ZTS, Zhaotong goat; GZS, Guizhou White goat; LNS, Liaoning Cashmere goat; CDM, Chengdu Brown goat; LLY, Longlin goat; LZS, Leizhou goat; MGR, Inner Mongolia Cashmere goat; CDS, Chaidamu goat; XJS, Xinjiang goat; XZS, Tibetan goat; ZWS, Zhongwei goat; SNB, Shannan White goat; HWS, Huanghuai goat; JNQ, Jining Gray goat; YMH, Yimeng Black goat; LBB, Lubei White goat; THS, Taihang goat; CJB, Yangtse River Delta White goat; MTS, Matou goat; YCB, Yichang White goat; XDH, Xiangdong Black goat; FQS, Fuqing goat; DYS, Daiyun goat.

**Table 2:** Analyses of conservation priorities for Chinese indigenous goat breeds

Breeds1)	WEDs	Bootstrap	WLM	PC_He_	PC_Weitz_	PC_Fst_	PC_5:1_
LLS	0	0	0	−0.047	2.81	0.349	2.333
MGS	0.0243	0.0211	0	−0.007	3.76	0.515	3.131
YLS	0.0073	0.0143	0.0419	−0.035	1.58	0.189	1.310
ZTS	0	0	0	−0.098	1.96	0.187	1.616
GZS	0	0	0	−0.161	2.67	0.232	2.197
LNS	0.1361	0.1324	0.1264	0.258	3.71	0.736	3.133
CDM	0.0048	0.0063	0.0242	0.121	6.71	1.034	5.610
LLY	0	0	0	−0.047	3.71	0.473	3.083
LZS	0	0	0	−0.148	6.57	0.783	5.448
MGR	0.1622	0.1613	0.1071	0.307	7.00	1.235	5.882
CDS	0.0229	0.0227	0.0964	0.173	2.81	0.538	2.370
XJS	0	0	0	0.071	2.86	0.457	2.394
XZS	0.0352	0.0255	0.0406	0.227	5.45	0.951	4.578
ZWS	0.0877	0.0972	0.0781	0.074	2.79	0.450	2.336
SNB	0	0	0	0.086	3.66	0.581	3.063
HWS	0.2017	0.2027	0.0853	0.223	5.6	0.969	4.702
JNQ	0.1488	0.1469	0.2621	0.363	2.21	0.619	1.902
YMH	0	0	0	0.035	2.04	0.313	1.705
LBB	0.1353	0.1296	0.1007	0.182	4.56	0.789	3.829
THS	0	0	0.0181	−0.014	1.94	0.256	1.614
CJB	0	0	0	−0.071	3.34	0.402	2.770
MTS	0	0	0	−0.195	3.49	0.316	2.875
YCB	0	0	0	−0.124	4.14	0.467	3.428
XDH	0	0	0	−0.218	2.52	0.161	2.063
FQS	0.0337	0.04	0.0193	−0.200	2.67	0.198	2.191
DYS	0	0	0	−0.207	3.49	0.305	2.873

Contribution made by each breed to total genetic diversity for 26 Chinese indigenous goat breeds based on methods.

MEK, marker-estimated kinships; WEDs, which vary based on weighted equal drift similarity; Bootstrap, WEDS with bootstrap procedure; WLM, weighted log-linear model; PC_weitz_, Weitzman approach; PC_He_, proportion of expected heterozygosity; PC_Fst_, aggregate methods based on Fst; and PC_5:1_, the Piyasation and Kinghorn formula. Values representing high contributions to genetic diversity are shown in boldface.

1) LLS, Longling yellow goat; MGS, Maguan poll goat; YLS, Yuling goat; ZTS, Zhaotong goat; GZS, Guizhou White goat; LNS, Liaoning Cashmere goat; CDM, Chengdu Brown goat; LLY, Longlin goat; LZS, Leizhou goat; MGR, Inner Mongolia Cashmere goat; CDS, Chaidamu goat; XJS, Xinjiang goat; XZS, Tibetan goat; ZWS, Zhongwei goat; SNB, Shannan White goat; HWS, Huanghuai goat; JNQ, Jining Gray goat; YMH, Yimeng Black goat; LBB, Lubei White goat; THS, Taihang goat; CJB, Yangtse River Delta White goat; MTS, Matou goat; YCB, Yichang White goat; XDH, Xiangdong Black goat; FQS, Fuqing goat; DYS, Daiyun goat.

**Table 3:** Contribution by Chinese goat breeds to total diversity, based on Cabalero and Toro [9]1)

Breed2)	*f*_ii_	D_Nei_	Contribution to f	Contribution to D	GDT|i	Loss/gain (%)	PC1 (%)	PC2 (%)
LLS	0.4165	0.1232	0.0108	0.0272	0.7408	0	3.673	3.673
MGS	0.4078	0.1218	0.0102	0.0264	0.7406	0	3.565	3.700
YLS	0.3781	0.1053	0.0091	0.0253	0.7408	0	3.417	3.767
ZTS	0.4130	0.1166	0.0105	0.0260	0.7412	0.1	3.511	3.646
GZS	0.4236	0.1156	0.0110	0.0256	0.7417	0.2	3.457	3.592
LNS	0.3439	0.1123	0.0093	0.0330	0.7386	−0.3	4.456	3.997
CDM	0.4001	0.1279	0.0111	0.0313	0.7396	−0.1	4.227	3.781
LLY	0.3678	0.0990	0.0099	0.0282	0.7409	0	3.808	3.794
LZS	0.4233	0.1177	0.0121	0.0288	0.7416	0.1	3.889	3.605
MGR	0.3442	0.1222	0.0074	0.0276	0.7382	−0.3	3.727	4.037
CDS	0.2967	0.0829	0.0086	0.0343	0.7392	−0.2	4.632	4.078
XJS	0.3159	0.0841	0.0094	0.0335	0.7400	−0.1	4.524	3.983
XZS	0.3217	0.1014	0.0081	0.0306	0.7388	−0.2	4.132	4.051
ZWS	0.3217	0.0961	0.0040	0.0143	0.7400	−0.1	1.931	4.010
SNB	0.3218	0.0884	0.0090	0.0318	0.7399	−0.1	4.294	3.983
HWS	0.2780	0.0808	0.0067	0.0297	0.7389	−0.2	4.011	4.172
JNQ	0.2692	0.0840	0.0073	0.0356	0.7378	−0.4	4.808	4.240
YMH	0.3038	0.0752	0.0083	0.0303	0.7403	0	4.092	4.010
LBB	0.3011	0.0889	0.0071	0.0280	0.7392	−0.2	3.781	4.091
THS	0.3486	0.0928	0.0107	0.0331	0.7406	0	4.470	3.875
CJB	0.3641	0.0940	0.0081	0.0227	0.7411	0.1	3.065	3.794
MTS	0.3806	0.0946	0.0120	0.0317	0.7419	0.2	4.281	3.713
YCB	0.3522	0.0849	0.0098	0.0282	0.7414	0.1	3.808	3.808
XDH	0.3976	0.0986	0.0110	0.0270	0.7421	0.2	3.646	3.646
FQS	0.4361	0.1197	0.0123	0.0278	0.7420	0.2	3.754	3.551
DYS	0.4985	0.1466	0.0119	0.0226	0.7420	0.2	3.052	3.362

*f*_ii_, average co-ancestries; D_Nei_, Nei’s genetic distance; f, contribution to global co-ancestry; D, absolute contribution to the total genetic diversity; GDT|i, global diversity; loss/gain(%), the % loss/gain after removing a population from the pool; PC, proportional contribution to gene diversity; PC1 estimates are weighted by population size; PC2 estimates ignore sample size.

1) Values representing high contributions are shown in boldface. Mean co-ancestry within-breed, *f* = 0.363; mean Nei’s minimum distance in the metapopulation, D = 0.103; mean co-ancestry in the metapopulation, *f* = 0.246; global genetic diversity of the metapopulation, GDT = 0.741.

2) LLS, Longling yellow goat; MGS, Maguan poll goat; YLS, Yuling goat; ZTS, Zhaotong goat; GZS, Guizhou White goat; LNS, Liaoning Cashmere goat; CDM, Chengdu Brown goat; LLY, Longlin goat; LZS, Leizhou goat; MGR, Inner Mongolia Cashmere goat; CDS, Chaidamu goat; XJS, Xinjiang goat; XZS, Tibetan goat; ZWS, Zhongwei goat; SNB, Shannan White goat; HWS, Huanghuai goat; JNQ, Jining Gray goat; YMH, Yimeng Black goat; LBB, Lubei White goat; THS, Taihang goat; CJB, Yangtse River Delta White goat; MTS, Matou goat; YCB, Yichang White goat; XDH, Xiangdong Black goat; FQS, Fuqing goat; DYS, Daiyun goat.

**Table 4:** Pairwise correlation coefficients between contributions obtained with different methods

Items	WEDs	Bootstrap	WLM	PC_He_	PC_Weitz_	PC_Fst_	PC_5.1_	PC1
Bootstrap	0.998	-	-	-	-	-	-	-
WLM	0.799	0.797	-	-	-	-	-	-
PCHe	0.742	0.726	0.781	-	-	-	-	-
PCweitz	0.344	0.327	0.060	0.321	-	-	-	-
PCFst	0.620	0.599	0.435	0.732	0.880	-	-	-
PC5.1	0.358	0.341	0.077	0.341	1.000	0.890	-	-
PC1	0.084	0.057	0.283	0.391	0.081	0.255	0.090	-
PC2	0.654	0.643	0.687	0.887	0.137	0.544	0.156	0.398

Method acronyms are defined in Tables 2 and 3.

WEDs, which vary based on weighted equal drift similarity; Bootstrap, WEDS with bootstrap procedure; WLM, weighted log-linear model; PC_He_, proportion of expected heterozygosity; PC_weitz_, Weitzman approach; PC_Fst_, aggregate methods based on Fst; and PC_5:1_, the Piyasation and Kinghorn formula. PC, proportional contribution to gene diversity; PC1 estimates are weighted by population size; PC2 estimates ignore sample size.
