# Supplementary material for: Insights into the genetic diversity of indigenous goats and their conservation priorities
Source: Asian-Australas J Anim Sci. 2019 Feb 9;32(10):1501–10. doi: 10.5713/ajas.18.0737 (PMC6718908; doi:10.5713/ajas.18.0737)
Supplement: Supplementary file 1 [file ajas-18-0737-suppl.pdf]

Table S1: Observed (N_A) and effective (N_E) number of alleles, observed (H_O) and expected (H_E) heterozygosities and F-statistics (F_{IS}, F_{ST} AND F_{IT}) across the 26 goat breeds.

Locus	N_A	N_E	H_O	H_E	F_{IS}	F_{IT}	F_{ST}
CSRD247	6.923±0.396	4.037±0.281	0.65±0.034	0.714±0.023	0.090	0.200	0.120
ETH10	2.923±0.214	1.575±0.107	0.357±0.069	0.291±0.045	-0.224	0.043	0.218
MAF209	2.308±0.092	1.393±0.06	0.269±0.041	0.25±0.03	-0.074	0.108	0.169
OARAE54	7.346±0.47	2.866±0.231	0.59±0.031	0.604±0.026	0.023	0.186	0.167
SRCRSP15	4.923±0.207	2.539±0.14	0.542±0.023	0.576±0.022	0.060	0.192	0.140
SRCRSP3	4.615±0.215	2.675±0.158	0.528±0.032	0.586±0.028	0.100	0.262	0.180
SRCRSP5	7.077±0.376	3.732±0.262	0.677±0.03	0.684±0.028	0.010	0.147	0.139
TGLA53	6.423±0.434	2.95±0.253	0.384±0.038	0.602±0.031	0.362	0.460	0.153
DRBP1	7.615±0.464	4.265±0.217	0.362±0.024	0.75±0.013	0.517	0.566	0.101
ILSTS087	7.769±0.279	4.784±0.304	0.721±0.026	0.764±0.019	0.056	0.155	0.105
INRABERN172	5.077±0.199	2.701±0.125	0.576±0.03	0.608±0.02	0.052	0.132	0.085
MAF065	7.192±0.451	3.947±0.213	0.71±0.022	0.728±0.014	0.025	0.104	0.081
MCM527	5.808±0.328	2.812±0.17	0.609±0.032	0.614±0.021	0.008	0.089	0.082
OARFCB20	6.154±0.264	3.206±0.184	0.595±0.043	0.65±0.028	0.085	0.211	0.138
SPS113	6.115±0.262	3.469±0.2	0.673±0.025	0.683±0.022	0.013	0.122	0.110
SRCRSP8	6.808±0.38	4.053±0.233	0.554±0.049	0.733±0.015	0.244	0.332	0.116
ILSTS029	7.154±0.609	3.274±0.312	0.653±0.036	0.624±0.032	-0.046	0.153	0.190
INRA023	5.923±0.517	3.224±0.304	0.506±0.05	0.62±0.031	0.183	0.413	0.281
INRA063	6.346±0.415	3.384±0.304	0.415±0.04	0.642±0.031	0.354	0.493	0.215
INRABERN185	4.000±0.222	2.075±0.117	0.469±0.036	0.487±0.023	0.037	0.309	0.282
P19(DYA)	6.577±0.416	3.482±0.261	0.636±0.027	0.674±0.023	0.057	0.170	0.120
SRCRSP23	7.654±0.61	3.721±0.333	0.709±0.032	0.676±0.028	-0.048	0.120	0.160
SRCRSP9	7.577±0.315	4.002±0.178	0.724±0.045	0.738±0.011	0.019	0.140	0.123
TCRVB6	8.731±0.406	3.858±0.302	0.551±0.033	0.705±0.02	0.218	0.309	0.116
BM6444	12.385±0.60	6.202±0.299	0.564±0.037	0.827±0.01	0.319	0.382	0.093
ILSTS011	6.385±0.249	3.306±0.173	0.669±0.035	0.675±0.019	0.009	0.104	0.096
ILSTS005	4.769±0.231	2.827±0.119	0.666±0.03	0.63±0.016	-0.057	0.062	0.113
SRCRSP7	3.846±0.205	2.517±0.13	0.472±0.045	0.571±0.026	0.174	0.285	0.135
OARFCB48	6.462±0.397	3.931±0.279	0.785±0.031	0.716±0.018	-0.096	0.010	0.097
MAF70	7.115±0.339	3.746±0.237	0.707±0.027	0.706±0.018	0.000	0.111	0.111
Mean	6.333±0.094	3.352±0.053	0.577±0.008	0.638±0.006	0.082±0.029	0.212±0.026	0.141±0.010

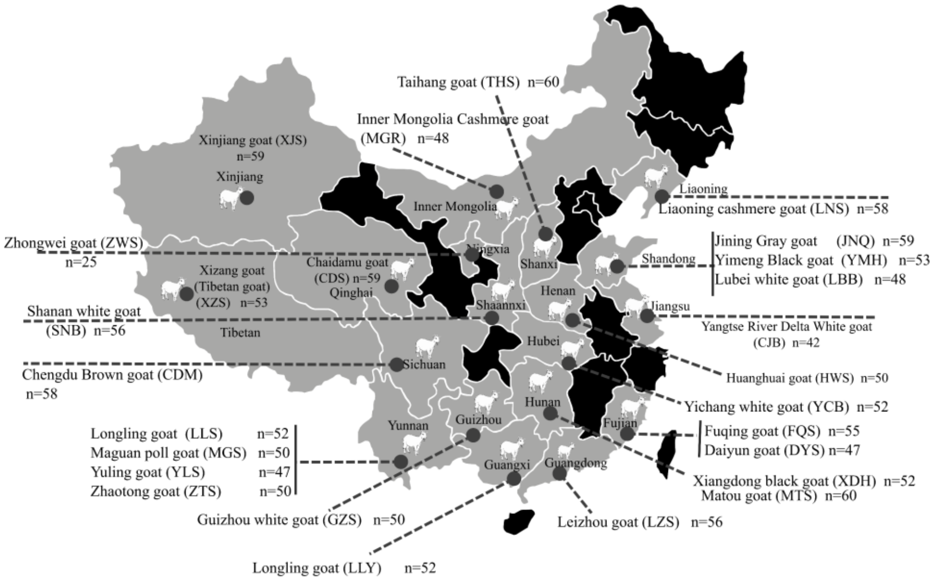

Supplementary Figure S1. The distribution of the 26 Chinese indigenous goat populations.

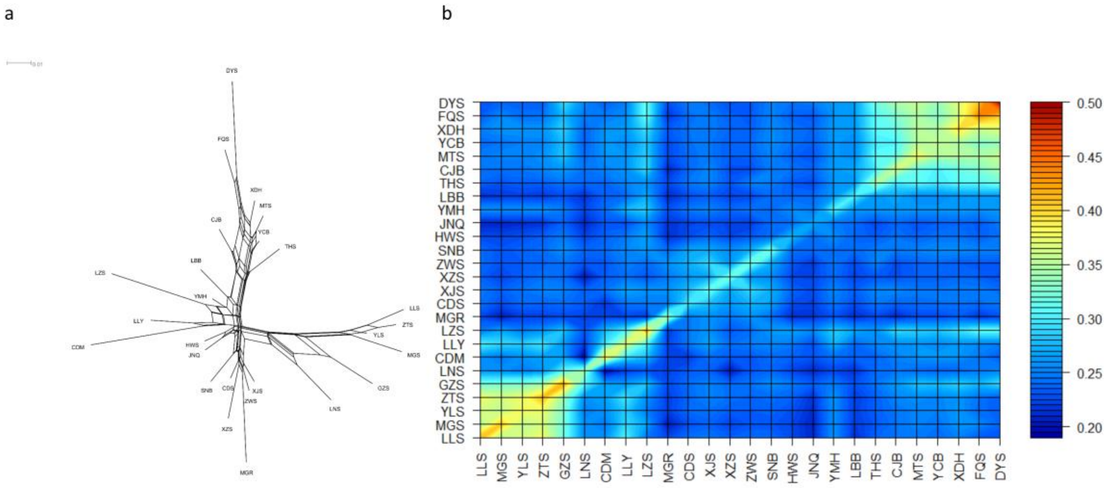

Supplementary Figure S2. (a) Neighbor-net graph of Reynolds' distance. (b) Contour plots of average co-ancestries (f_m). Breed acronyms are defined in Figure 1.
